# Hyperglycemia and nocturnal systolic blood pressure are associatedwith left ventricular hypertrophy and diastolic dysfunction in hypertensive diabetic patients

**DOI:** 10.1186/1475-2840-5-19

**Published:** 2006-09-12

**Authors:** João S Felício, Juliana T Pacheco, Sandra R Ferreira, Frida Plavnik, Valdir A Moisés, Oswaldo Kohlmann, Artur B Ribeiro, Maria T Zanella

**Affiliations:** 1 Endocrinology Division, Universidade Federal do Pará, Belém, Brazil; 2 Epidemiology Division, Universidade Estadual de São Paulo, São Paulo, Brazil; 3 Nephrology and Endocrinology Divisions, UNIFESP, Universidade Federal de São Paulo, São Paulo, Brazil

## Abstract

**Background:**

The aim of this study was to determine if hypertensive type 2 diabetic patients, when compared to patients with essential hypertension have an increased left ventricular mass index (LVMI) and a worse diastolic function, and if this fact would be related to 24-h pressoric levels changes.

**Methods:**

Ninety-one hypertensive patients with type 2 diabetes mellitus (DM) (group-1 [G1]), 59 essential hypertensive patients (group-2 [G2]) and 26 healthy controls (group-3 [G3]) were submitted to 24-h Ambulatory Blood Pressure Monitoring (ABPM) and echocardiography (ECHO) with Doppler. We calculated an average of fasting blood glucose (AFBG) values of G1 from the previous 4.2 years and a glycemic control index (GCI) (percentual of FBG above 200 mg/dl).

**Results:**

G1 and G2 did not differ on average of diurnal systolic and diastolic BP. However, G1 presented worse diastolic function and a higher average of nocturnal systolic BP (NSBP) and LVMI (NSBP = 132 ± 18 vs 124 ± 14 mmHg; P < 0.05 and LVMI = 103 ± 27 vs 89 ± 17 g/m^2^; P < 0.05, respectively). In G1, LVMI correlated with NSBP (r = 0.37; P < 0.001) and GCI (r = 0.29; P < 0.05) while NSBP correlated with GCI (r = 0.27; P < 0.05) and AFBG (r = 0.30; P < 0.01). When G1 was divided in tertiles according to NSBP, the subgroup with NSBP≥140 mmHg showed a higher risk of LVH. Diabetics with NSBP≥140 mmHg and AFBG>165 mg/dl showed an additional risk of LVH (P < 0.05; odds ratio = 11). In multivariate regression, both GCI and NSBP were independent predictors of LVMI in G1.

**Conclusion:**

This study suggests that hyperglycemia and higher NSBP levels should be responsible for an increased prevalence of LVH in hypertensive patients with Type 2 DM.

## Background

Clinical, epidemiologic and pathological data support the occurrence of a specific cardiomyopathy related to diabetes mellitus (DM) [1-3]. However, the exact cause of this complication is still being discussed. Proposed causes include metabolic abnormalities (hyperglycemia and changes in myocardial lipid metabolism), hypertension and autonomic neuropathy [3-6]. As DM is usually associated to hypertension and coronary arteriosclerosis [2] and they all can reduce myocardial performance, it's hard to dissociate cardiac abnormalities (LVH and diastolic dysfunction) originated from those conditions to that straightly related to metabolic changes of DM.

In 1992, Grossman et al [7], showed that hypertensive patients with DM, when compared to essential hypertensive patients, had a higher LVMI independent of office blood pressure. The principal determinant of the BP circadian pattern appears to be the sympathetic nervous system and DM has been associated with elevated levels of nocturnal BP [8]. We have demonstrated that the improvement of glycemic control may play an independent role in the reversal of LVH in hypertensive patients with type 2 DM [9]. Nevertheless, in our study, 24-h BP levels were not evaluated.

The purpose of this study was to determine if hypertensive type 2 diabetic patients, when compared to patients with essential hypertension have an increased LVMI and a worse diastolic function, and if this fact would be related to 24-h pressoric levels changes.

## Methods

### Patients

A total of 176 subjects recruited from our Hypertension Clinic during one year period were enrolled in this study. The 91 (64 women and 27 men) hypertensive patients with type 2 DM (group-1 [G1]), the 59 (46 women and 13 men) nondiabetic hypertensive patients (group-2 [G2]) and the 26 healthy control subjects (group-3 [G3]) were submitted to 24-h ABPM and to ECHO with Doppler to evaluate LVMI and diastolic function. ABPM was done after a 15 days washout of all antihypertensive drugs in order to evaluate patient's real blood pressure and minimize the effect of BP variations during the long period of cardiac hypertrophy development. Ten patients of G1 couldn't have their LVMI measured because of technical problems (narrow window: obesity); the other 81 remainders were divided in 3 subgroups (tertiles) based on their NSBP to stablish the prevalence of LVH. G1 had the following parameters calculated: the average of fasting blood glucose (AFBG) values and the glycemic control index (GCI).

All patients had normal levels of serum creatinine and only two had abnormal 24-h urinary protein excretion (>150 mg/24h). Patients with clinical or echocardiographic evidence of ischemic or valvular heart disease were not included in this study, nor were those with congestive heart failure, alcoholism or secondary or severe hypertension. Criteria for established hypertension were systolic and diastolic blood pressure≥140/90 mmHg on repeated measurements [10]. Diabetes was diagnosed according to the standart criteria [11]. Type 2 DM were indentified as those with disease onset at the age of 30 years or after with no need of insulin treatment. They were treated only with diet or diet plus oral antidiabetic agents (sulphonylureas, metformin, or acarbose) and antihypertensive drugs. None of them was treated with insulin during the study. This study was approved by the Institutional Ethics Committee.

### Echocardiography

M-mode, two-dimensional echocardiographic and cardiac Doppler studies were performed using a commercially available echo-Doppler unit (Esaote Biomedica, Florence, Italy; model SIM 5000) equipped with a 2.5-MHz mechanical transducer. It was performed with patients in the partial left lateral supine position. M-mode measurements were performed acording to the recommendations of the American Society of Echocardiography [12]. Left ventricular mass (LVM) was calculated as previously recommended by Deveraux et al [13]. The LVMI was calculated by dividing LVM by the body surface area. LVH was present if LVMI was ≥134 g/m^2 ^in men and ≥110 g/m^2 ^in women [13,14]. All examinations were analyzed blindly by one independent echocardiographer. Transmitral blood flow signals were obtained on top of mitral valve by apical 4-chamber-view and the following measurements were made on consecutive cardiac cycles: (1) peak flow velocity of early left ventricular (LV) filling (peak E), (2) peak flow velocity of late (atrial) LV filling (peak A), (3) early deceleration time (DT), (4) isovolumic relaxation period (IVR) and (5) the ratio between early and late diastolic flow velocity peaks (E/A ratio) (normal values: peak E>50 cm/s; peak A<80 cm/s; DT<240 ms; IVR<110 ms, and E/A ratio≥1) [15,16]. All measurements of diastolic function were done with normal heart rate (60–100 bpm).

### Ambulatory Blood Pressure Monitoring

The 24-hour ABPM recording was performed using a SpaceLabs – 90207 automatic cuff-oscillometric device (Spacelabs, Inc. Redmond, WA – USA) after 15 days washout of all antihypertensive drugs. Patients should keep their habitual routine and present a report with the activities done. Systolic and diastolic BP and heart rate were recorded at 15-minutes intervals during daytime and nighttime periods. Daytime period included all activities done from 8am to 8pm and the 8pm to 8am period was considered nighttime. After this, the average of systolic and diastolic BP was calculated for each hour, for daytime, nighttime and for the 24-hour period. The exam was considered reliable when at least 75% of the measurements were successfully executed. Moreover, it was calculated the nocturnal BP decrease ([diurnal systolic blood pressure - nocturnal systolic blood pressure] × 100/diurnal systolic blood pressure). It was considered normal values of nocturnal BP decrease greater than 10% (dippers) and patients that showed BP decrease lower than this value were called "nondippers". We used the absolute values of NSBP instead of nocturnal BP fall to divide the subgroups in tertiles because the first variable has a minor coefficient of variation (data not published).

### Other measurements

To create a measure of the previous long-term glycemic control, the average of all fasting blood glucose (AFBG) values available before the study was calculated (mean period of 4.2 years and mean of 3.7 values of FBG/patient/year). A total of 1014 FBG were evaluated. If several FBG were recorded during a month, only the first value of the month was used. These FBG values were also used to calculate the glycemic control index (GCI) (percentual of FBG values above 200 mg/dl).

### Statistical analysis

All normally distributed values were given as mean ± SD and all other values were given as median (range). In comparisons of the nonnormally distributed variables, the Kruskal-Wallis test on variance was used to test the differences between the three groups. If differences were found, the Mann-Whitney test was used for comparisons between two groups. For all normally distributed variables, analysis of variance was performed to test the differences among the three groups. If differences were found, the Student's *t *test was used for comparison between two groups. Fisher's exact or chi-squared tests for trend have been used to compare categorical data. For correlation analysis, correlation coefficients (Pearson or Spearman) were calculated. For regression analysis (forward stepwise regression), in a first model, the LVMI was used as a dependent variable and age, sex, body mass index (BMI), NSBP, GCI and diabetes and hypertension duration were used as independent variables. A second model of regression analysis was used with NSBP as dependent variable and age, BMI, AFBG and DM and hypertension duration as independent variables. A P value (two-tailed) less than 0.05 was considered statistically significant. All calculations were made with a commercially available program, SigmaStat 1.0 (Jandel Scientific Corporation, Chicago, Illinois).

## Results

Clinical characteristics of the three groups are in table 1. In G1, the AFBG was 156 ± 41 mg/dl and duration of diabetes was 48 months (5 – 456). We also evaluated the antihypertensive drugs used during the year before the study. We found that diabetics, when compared to essential hypertensives, used more frequently ACE-inhibitors (30% vs 13%; P < 0.05; odds ratio = 2.9) and calcium antagonists (46% vs 15%; P < 0.05; odds ratio = 4.7) than others antihypertensive drugs (diuretics, beta-blockers, etc) (24% vs 72%; P < 0.05; odds ratio = 8.0).

**Table 1:** Clinical characteristics of study population

**Group**	**Sex (M/F)**	**Age (years)**	**BMI (kg/m^2^)**	**Duration of EH (months)**	**DSBP (mmHg)**	**DDBP (mmHg)**
G1	27/64	57 ± 9	28 ± 4	144 (3 – 612)	146 ± 19	90 ± 12
G2	13/46	54 ± 10	27 ± 4	120 (1 – 420)	140 ± 13	90 ± 9
G3	11/15	55 ± 8	27 ± 5	-	122 ± 10*	77 ± 8*

*P < 0.05 G3 vs G1 and G2

DDBP = diurnal diastolic BP; DSBP = diurnal systolic BP; EH = essential hypertension

G1 and G2 did not differ in age, BMI, sex, duration of hypertension and average of diurnal systolic and diastolic BP, however healthy control subjects (G3) differed in diurnal BP levels when compared to G1 and G2 (table 1). There was no difference between office BP of diabetic hypertensive patients and nondiabetic hypertensive patients as well.

According to table 2, G1 presented higher NSBP and LVMI. Patients with DM also presented a worse diastolic function (DT and peak A) (table 2). Additionally, when compared G1 and G2, the first group showed more subjects with abnormal values of DT (45% vs 15%; P < 0.001; odds ratio = 3.4) and peak A (40% vs 16%; P < 0.05; odds ratio = 2.6).

**Table 2:** Nocturnal BP recording and ECHO with Doppler results in study population

**Group**	**G1**	**G2**	**G3**
N	91	59	26
NSBP (mmHg)	132 ± 18‡	124 ± 14	108 ± 11
NDBP (mmHg)	77 ± 10*	75 ± 98*	64 ± 8
LVMI (g/m^2^)	103 ± 27*†	89 ± 17	82 ± 14
LVH (%)	26*	14	0
DT (ms)	236 ± 58*†	198 ± 49	199 ± 34
IVR (ms)	98 ± 20*	101 ± 27*	83 ± 17
E/A ratio	0.84 (0.3 – 20.2)*†	1.0 (0.5 – 1.8)	1.1 (0.7 – 1.8)

‡ P < 0.05 between the three groups; * P < 0.05 vs G3; † P < 0.05 vs G2

NDBP = nocturnal diastolic BP

In G1, LVMI correlated to NSBP (r = 0.37, P < 0.01) and GCI (r = 0.29, P < 0.05), while NSBP correlated to GCI (r = 0.27, P < 0.05) and AFBG (r = 0.30, P < 0.01).

**Figure 1:**
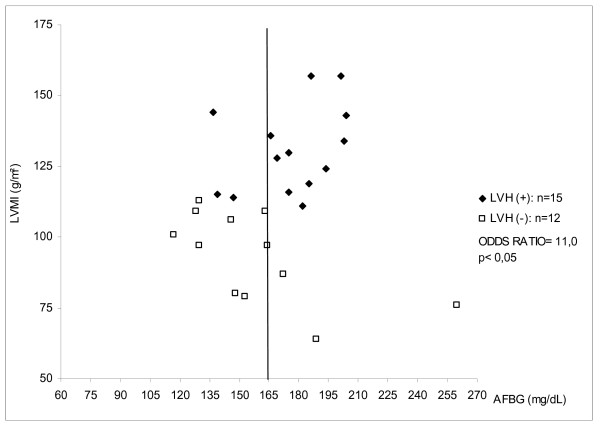
Risk of left ventricular hypertrophy and average of fasting blood glucose values in the subgroup with nocturnal systolic blood pressure ≥140 mmHg. LVMI – left ventricular mass index AFBG – average of fasting blood glucose LVH – left ventricular hypertrophy

When G1 was divided in tertiles according to NSBP (table 3), the subgroup with NSBP≥140 mmHg showed a higher risk of LVH (P < 0.001, odds ratio = 7.5 when compared to patients with NSBP≤124 mmHg and P < 0.01, odds ratio = 3.8 when compared to patients with NSBP between 124 and 140 mmHg). In the subgroup with NSBP≥140 mmHg, patients with LVH (n = 15) did not differ of patients with normal LVMI (n = 12), according to age, hypertension duration, NSBP and diurnal BP, however they presented a higher GCI (table 4). Furthermore, diabetics with NSBP≥140 mmHg and AFBG>165 mg/dl showed an additional risk of LVH (P < 0.05; odds ratio = 11) (figure 1).

**Table 3:** Clinical parameters of diabetic hypertensive patients (G1) based on their NSBP

**Group**	**NSBP≤124 mmHg**	**124< NSBP < 140**	**NSBP≥140 mmHg**
N	27	27	27
Age (years)	56 ± 11	58 ± 7	58 ± 8
BMI (kg/m^2^)	28 ± 5	28 ± 4	29 ± 4
DDBP (mmHg)	81 ± 8	88 ± 10	101 ± 9†
NDBP (mmHg)	67 ± 6	78 ± 6	87 ± 8†
DSBP (mmHg)	13 ± 28	143 ± 11	168 ± 17†
NSBP (mmHg)	115 ± 4	131 ± 4	154 ± 15†
SBPD (%)	12 ± 5	8 ± 7*	8 ± 6*
LVMI (g/m^2^)	93 ± 30	101 ± 23	114 ± 24*
LVH (yes/no)	2/25	4/23	15/12*‡
AFBG	150 ± 45	158 ± 38	168 ± 31

† P < 0.05 between the three groups; * P < 0.05 vs NSBP≤124 mmHg; ‡ P < 0.05 vs 124< NSBP < 140

DDBP = diurnal diastolic BP; DSBP = diurnal systolic BP; NDBP = nocturnal diastolic BP;

SBPD = systolic BP decrease

**Table 4:** Subgroup with NSBP≥140 mmHg divided according to the presence of LVH

**Subgroup**	**LVH +**	**LVH -**
N	15	12
Age (years)	57 ± 9	60 ± 7
BMI (kg/m^2^)	28 ± 4	31 ± 4
Hypertension duration (months)	192 (36 – 420)	126 (18 – 612)
DM duration (months)	108 (6 – 360)	54 (8 – 312)
DSBP (mmhg)	169 ± 12	166 ± 22
NSBP (mmHg)	152 ± 10	157 ± 20
DDBP (mmHg)	102 ± 8	100 ± 10
NDBP (mmHg)	86 ± 8	88 ± 8
GCI (%)	29 (4 – 67)*	8 (0–75)

*P < 0.05

In G1, the first model of multivariate regression showed both GCI and NSBP as independent predictors of LVMI (r^2 ^= 0.11; P < 0.01 and r^2 ^= 0.08; P < 0.01, respectively). In the second model, only hypertension duration was an independent predictor of NSBP (r^2 ^= 0.11, P < 0.01).

## Discussion

The present study demonstrated that hypertensive patients with type 2 DM when compared to patients with essential hypertension presented higher NSBP and LVMI. These findings occurred independently of sex, age, BMI and diurnal BP levels. We have also found a worse diastolic function in diabetic patients.

Grossman et al [7], comparing hypertensive type 2 DM patients with essential hypertensives also found a higher LVMI in the first group. Nevertheless, they didn't evaluate the 24-h BP levels. We have confirmed these data and have showed that in hypertensives with type 2 DM, the NSBP could be a new important co-factor, that in addition to hyperglycemia, would be responsible for a higher prevalence of LVH in these patients.

Palmieri et al [17], evaluating almost 400 hypertensive diabetic patients in comparison with essential hypertensive, have found that the first group had higher LVM independently of BP levels. Furthermore, Tenenbaum et al [18] also described that type 2 DM was associated with a higher prevalence of LVH in hypertensive women but not in men. Finally, Galderisi et al [19], evaluating 1986 men and 1519 women from the original Framingham Study cohort, in a multivariate model, concluded that DM is an independent contributor to LVM. Nevertheless, all these authors have not performed ABPM in their studies.

There are several mechanisms that could be responsible for a lower fall of BP during sleep. Some authors have associated it to diabetic nephropathy (DN) [20] that could be explained by an increase of extracellular fluid [21]. In our study, only 2 of 91 diabetics had abnormal proteinuria and all of them had normal creatinine levels. Therefore, DN couldn't justify elevated NSBP in our group. According to Rutter et al [22], LVH is more common and severe in those with microalbuminuria and its presence may be related to raised night/day systolic blood pressure ratio. Nevertheless we have not done microalbuminuria in our patients.

Patients with type 2 DM are more stable in blood glucose control than patients with type 1 DM [23] and FBG has been commonly used to monitor glycemic control in type 2 diabetic patients treated with diet alone or oral hypoglycemic agents [24]. In both cross-sectional [25-27] and prospective [28] studies a good correlation was shown between FBG and HbA1. In addition, the retrospective average of FBG values is considered a good index to establish a previous long-term glycemic control in patients with type 2 DM [29]. Hence, it is unlikely that the lack of HbA1 values to evaluate previous glycemic control could have influenced our results.

It has also been suggested that a poor glycemic control could be responsible for the increase of NSBP levels [30]. In fact, in our study, we found a positive correlation between these two variables. However, in our models of multiple regression, only hypertension duration was an independent predictor to NSBP. Furthermore, we found that both hyperglycemia and NSBP were independent predictors to LVMI. These findings support the hypothesis that hyperglycemia could not be directly responsible for the increased NSBP in diabetic patients, although it could have influenced on this parameter.

Autonomic neuropathy (AN) could be a via through hyperglycemia would elevate NSBP levels. Some studies have related AN to an increase of LVM [31] and to the loss of nocturnal BP decrease [8]. In addition, we compared normotensive diabetic patients with normal subjects and found that a higher score of AN was already correlated to a higher LVMI (data not published). AN could reduce the nocturnal BP falls through the reduction of vagal tonus and increase of cardiac output during sleep [21].

It has been described a correlation between hyperglycemia and LVMI in diabetic patients [4,7] and some authors have suggested mechanisms to explain how hyperglycemia acts on cardiac mass [4]. Nevertheless, prospective data evaluating the effect of a better glycemic control on LVM are rare [9]. The retrospective average of FBG values is considered a good index to establish a previous long-term glycemic control in patients with type 2 DM [29]. Coutinho et al [32] have recently published a meta-regression analysis of 20 studies including 95,783 nondiabetic individuals who had 3,707 cardiovascular events and who were followed for 12.4 years. They showed that high fasting glucose values increased the risk for cardiovascular events. Fasting glucose of 109 mg/dl increased the risk of cardiovascular events by 1.33 compared with a fasting glucose of 75 mg/dl. Therefore, it is obvious that glucose level seems to be a risk factor for cardiovascular events even within a range that is below the diabetic threshold. Glucose is likely to be a continuous cardiovascular risk factor, similar to cholesterol and blood pressure. This study showed that diabetic patients with NSBP≥140 mmHg had a great increase on prevalence of LVH. In this subgroup, when the average of FBG was >165 mg/dl, the risk of LVH had an additional increment. These high values of FBG, that started to increase the risk of LVH, could be explained by the longer diabetes duration. Furthermore, Staessen et al [33], in a meta-analysis of 23 studies, including a total of 3,476 normal subjects, concluded that only levels ≥137 mmHg for NSBP should be considered as definite hypertension. This NSBP value is too close to the level that we found to start increasing the risk of LVH.

The coexistence of LVH and possible ischemic heart disease is a well-known phenomenon in general population and it has been suggested that ischemic heart disease is a consequence rather than a cause of LVH [34]. In addition, the findings of Ghali et al [35] and Fujita et al [36] suggest that the contribution of ischemia to enlarge LVM is rather small. We have not excluded silent ischemia, a condition claimed to be frequently present in type 2 DM. Nevertheless, this claim is based on noninvasive methods revealing a prevalence of ischemia in 36% [37] and 31% [38] of patients with type 2 DM. In contrast, coronary angiography only revealed prevalence of significant coronary artery disease in 8% and 11% of the diabetic patients included in the two above-mentioned studies, respectively. Therefore, ischemic heart disease does not seem to be an important contributor to the LVH in our patients.

It has been suggested that abnormalities in lipid metabolism could be involved in the pathogenesis of diabetic cardiomyopathy [5]. Nevertheless, we have recently demonstrated that, in hypertensive patients with type 2 DM, the reversal of LVH can occur independently of any variation on lipids levels [9].

Almost all of our diabetic patients were using anti-hypertensive drugs for more than one year before the study. Most of them were in use of ACE-inhibitors and calcium antagonists, which are recognized efficients at LVH regression in hypertensive patients [39]. Hence, the kind of antihypertensive used can not justify the higher LVMI in the diabetic group.

Salmasi et al [40] described that in hypertensive patients, left ventricular diastolic function is determined by LMVI and the status of preclinical glucose intolerance. Our study found a strong relation between NSBP and LVMI and no relation between NSBP and diastolic function, what suggests that the worse diastolic function in our diabetic hypertensive patients could be caused by a higher LVMI. Some authors have also found a worse diastolic function in diabetics and some of them considering these abnormalities as largely irreversible [3,6,41]. Although we have not observed a correlation between NSBP and diastolic function, a intensive control of nocturnal pressoric overload might contribute to an improvement of this function through a possible reversal of LVH.

Aepfelbacher et al [42] have described interventricular septal thickness decrease and left ventricular mass regression in type 1 diabetic patients with improved glycemic control. They have not found any BP variation evaluated by ABPM during their study. Nevertheless, a small number of patients were evaluated and LVM was also not adjusted by body surface area to calculate left ventricular mass index, that is the best parameter to evaluate cardiac hypertrophy. Moreover, type 2 diabetic patients also present other confounders (lipid levels, aterosclerosis disease, arterial hypertension, obesity) that may influence the results.

A BP overload, detectable by ambulatory recording and not by clinic measurements, may be a major determinant of left ventricular diastolic dysfunction and left ventricular mass increase [43]. Our study reinforces the existence of a specific cardiomyopathy related to DM where higher NSBP levels and hyperglycemia should be co-factors for an increased prevalence of LVH. The benefits of an intensive control of NSBP in hypertensive patients with type 2 DM need to be clarified, what will be only possible if we include ABPM in the routine exams of all type 2 diabetic patients.

## Competing interests

The author(s) declare that they have no competing interests.

## Authors' contributions

JF conceived of the study, participated in its design and coordination and performed the statistical analysis. JP, SF, FP and OK participated in the sequence alignment. VM carried out the echocardiography. AR and MZ participated in the design of the study. All authors read and approved the final manuscript.
